# Andrews on the Physiology of the Three Registers of the Human Voice

**Published:** 1857-11

**Authors:** E. Andrews

**Affiliations:** Chicago, Ill.


### *Andrews on the Physiology of the three Registers of the Human Voice.*

At the request and with the assistance of Mr. C. M. Cady, Editor of the *Chicago Musical Review*, and well known talented ex-Editor of the *N. Y. Musical Review*, I have lately made some new investigations respecting the action of the vocal organs in singing, and have arrived at some conclusions which, so far as I know, are not to be found in any publication, and for which therefore I may claim originality.

Mr. Cady is a gentleman not only of high musical attainments, but also of liberal education, and of an unusually clear and analytic mind, so that with his assistance and suggestions I have been able to attain an explanation of facts which with most of our leading professors of music would have been impossible.

All educated singers understand that there are three modifications of the voice cultivated, which are called *registers*, and that in passing from one to the other the vocalist is conscious of some movement or change in the larynx.

The first, or the *Chest Register*, is that quality of voice produced by male singers on the lower and middle notes of their compass, and by the female on the lowest notes in her reach, for instance those below the middle C. This register is sonorous and capable of great power, and conveys to the ear a slight sense of roughness like the reedy tone of a clarionet. It is the tone adopted by the male in common conversation.

The second, or *medium register*, is that smooth mellow tone used by good tenor singers on the high notes and by females on the middle notes, for instance in the vicinity of G and A.

The third, or *head register*, is the clear flute like tone used by females on the high notes, for instance on the upper F and G. In the male voice the head register exists under the name of *falsetto*, but as it is not of good quality, the best authorities reject it as unworthy of cultivation in our sex, hence it is customary for musicians to say that the male voice has only two registers, the chest and the medium, while the female has three.—

Physiologically, however, the falsetto is the head register of the male, so that the three registers really exist in each sex. It may be proper to mention here for the benefit of those not familiar with musical terms, that the falsetto is that shrill forced tone capable of being uttered by the male, which resembles the female voice.

These three registers do not differ merely in *pitch.* They are different in quality. There are several parts of the scale which may be given in either of the chest or the medium register, and there are three others in the vicinity of C which a person can give at will in either the chest, the medium, or the head register, and in changing from one register to another on the same key, the singer is distinctly conscious of some action in the larynx.

It was the nature of this action which Mr. Cady requested me to investigate; the physiology of the whole matter being apparently unknown both to medical and musical writers.

The reader will pardon me if I here recapitulate briefly the anatomy of the larynx for the sake of perspicuity.

The frame work of the larynx is the cricoid cartilage which has the form of a ring. Upon the upper edge of this is articulated in front the thyroid cartilage, and posteriorly the two arytenoid cartilages. These three serve as pegs for the attachment of the vocal ligaments. The vocal ligaments consist of a pair of tendons attached to the thyroid cartilage anteriorly, and running one to each arytenoid cartilage posteriorly. Each one is connected to its own side of the larynx by a fold of mucous membrane. Thus each one has the form of a half moon with the tendon in its straight edge, and when they are put upon the stretch so as to bring the edges in contact, the two form a perfect diaphragm across the larynx. By this arrangement, therefore the motion of the thyroid cartilage forward and of the arytenoids backwards will put the ligaments upon the stretch across the larynx. The two arytenoid cartilages have the form of levers bent to a right angle. The fulcrum is at the angle where they are, articulated to the edge of the cricoid cartilage. The long arm of the lever projects upward into the cavity of the pharynx, and the short arm called the anterior process projects horizontally forward into the larynx; it is the short arm to which the vocal ligament is attached, *so that whatever motions the anterior process of the arytenoid cartilages make, the vocal ligaments will partake of.*

The arytenoid cartilages are so articulated as to allow of motion in all directions. Therefore, if the points of the anterior processes rotate outward, they will carry the edges of the two vocal ligaments asunder and leave a wide space for the passage of the breath, but if they are rotated inwards so as to be applied against each other, the vocal ligaments will have their edges brought into contact, and if the ligaments are put upon the stretch and air forced through the crevice between them, a vocal sound is produced.

It will be observed that the conditions under which the vocal ligaments act in uttering sound, are identical with those of the lips in blowing a sax horn or any of the trumpet instruments. In the latter operation, the lips are drawn tightly across the mouthpiece of the instrument and the breath driven through the crevice between them. In the larynx, the vocal ligaments are stretched across the cavity and applied to each other in the same manner. The ligaments however being thinner, give a more delicate sound than the lips. *The human vocal organs act on the principle of the trumpet.* It is important to be clear on this point, otherwise the subject of the register cannot be understood. That this is the method of vocal action, I have proved both by experiment and by *actually seeing the human vocal chords in action in the living subject.*

To try the experiment, take two slips of sheet india rubber and tie them tightly over the end of a tube, for instance the large end of a wooden stethoscope: let them be tightly stretched on, covering the whole end except a very minute fissure between their two edges. If you now put the small end to your mouth and blow, a clear musical sound will be produced, and the edges of the rubber may be distinctly seen to vibrate.

Some months ago, I saw the rare spectacle of the vocal ligaments exposed to sight in a living person.

A. B., in a temporary fit of insanity, cut his throat with a razor. The wound was of enormous size and, of course, gaped widely. The cutting instrument had shaved off the top of the larynx completely, just above the vocal ligaments, exposing them to full view. The patient had not severed any large vessels, and at the time I was called in, was free from delirium.

The arytenoid cartilages were seen on the posterior edge of the larynx, the superior processes pointing upward into the pharynx as usual, and the anterior processes rotated outwards so as to leave a wide angle between them, and the points rested against the sides of the laryngeal cavity. The vocal ligaments therefore were carried outward and folded against the alæ of the thyroid cartilage, so that the opening was large and the respiration silent and easy. The opportunity was too good to be lost, and at my request, the patient repeatedly uttered sounds while I watched the action of the organs. At every effort to speak the anterior processes of the arytenoids swung inwards, described about the eighth of a circle each, until they came in contact; this brought the vocal ligaments together; and they could now be distinctly seen stretched tightly over the whole opening like a diaphragm, and as the breath was expelled between them, the two coapted edges were easily observed to vibrate, while the sound was produced I repeated these observations until I was fully satisfied, and I regarded them with great interest, because such opportunities to see the vocal organs in action, must be extremely rare.

I consider therefore that I have clearly proved that the vocal organs act on the principle of the lips applied to the mouth piece of a trumpet, and not as Carpenter says, like the reed on a clarionett, still less as others have said, like a flute, like an æolian harp, like an organ pipe, or like a piano string.

The changes in pitch of the voice are accomplished as every one knows, by tightening or relaxing the vocal ligaments. This is effected by the action of muscles on the thyroid and arytenoid cartilages, to which the ligaments are attached. In the trumpet, similar changes of pitch are made by tightening and relaxing the lips. I have been thus prolix in prefacing this subject, because unless the foregoing points are borne in mind, the physiology of these organs cannot be understood.

The chest register, it will be borne in mind, is that peculiar quality of voice used by male singers on the low and middle notes, and by females on the low notes alone. It is reedy in character, conveying to the ear an impression of slight roughness, and giving a corresponding feeling in the larynx of the performer. It is accompanied by greater pressure of air in the chest than the other registers, as though the opening through which it is forced were smaller, and the resistance therefore greater. I suppose this quality of sound to be produced by *placing the edges of the vocal chords pretty firmly together*, so that they strike each other at every vibration. The proof of this proposition is first the analogy of other instruments, secondly the anatomy of the parts, and thirdly the sensations of the performer.

I have already shown that the larynx acts on the principle of the trumpet. Now if any one will experiment carefully with a sax horn or any similar instrument, he will find that like the human voice it has two registers corresponding to the chest and middle ; bnt that it has not the head register for reasons that will be apparent presently.

The quality in brass instruments corresponding to the chest register, when carried to excess, is that harsh rattling sound which players technically call "tearing the instrument." It is frequently heard in the trombone, especially when played by one who is ambitious to make more than his share of the noise. The chest register is used by brass bands in outdoor martial music, while the medium register is more used when inside of buildings and on soft passages.

If the experimenter carefully notices in what manner he produces these variations, he will find that in the chest register he presses his lips more firmly together, and is obliged to use a correspondingly greater pressure of the chest to force the air through them. Like causes produce like effects, and it is fair to conclude that in the chest register of the voice a similar pressure of the vocal chords against each takes place. If so, then we readily see why a greater force of the lungs is requisite in expelling the air.

If we recur to the anatomy of the parts, we find the thyroarytenoideus muscles adapted to this very function. They arise from the anterior part of the concavity of the thyroid cartilage just external to the vocal tendoms, and running backwards are attached to the external processes of the arytenoid cartilages, so that by their contraction they rotate the anterior processes inward, and press the ligaments together, and this action I witnessed in the case of the attempted suicide above mentioned.

Now if we consider the sensations of the vocalist, we are still further confirmed in the opinion that the chest resgister is the result of the contraction of the thyro-arytenoid muscles ; for in this register the singer is conscious of very great muscular effort in the larynx. On the high notes, this exertion becomes painful, hence the resort to the medium or head register for relief.

If we recur to the anatomy of the larynx, we find that the tension of the vocal chords on which the pitch depends, is regulated by the action the crico-thyroid muscles, which draw the thyroid cartilage forward thus tightening the chords.

Now the thyro-arytenoid muscles from their position tend to draw the thyroid cartilage backwards, hence these two pairs of muscles antagonize each other, so that when the thyro-arytenoids contract to rotate the arytenoid cartilages inwards and press the vocal chords together, they at the same time tend to relax the chords and lower the pitch of the sound, therefore the crico-arytenoid muscles in drawing the thyroid cartilage forward to raise the pitch, have to act not only against the physical tension of the chord, but also against the whole power of the thyro-arytenoid muscles. Hence to produce a high note in the chest register, causes a painful sense of muscular effort in the larynx, because the muscles which raise the pitch have to act against the whole power of the muscles which press the chords together.

Another proof that the chest register is the result of placing the edges of the chords in contact, is that the singer is conscious of a rough vibratory sensation as though the chords touched something.

Still another proof, and one which is perfectly decisive is obtained by the following simple experiment. Let one place his organs in position for a decided chest tone and then make the effort at expiration so gently as not to produce the sound. He will find that no air escapes, but that the passage of the larynx is perfectly closed showing that the vocal chords not only are in contact, but are pressed together with sufficient firmness to require some force to expel any air between them. If he now increase the force of the expiratory effort sufficiently to expel the breath, a marked chest tone is the result.

I think I have now given the physiology of this register with a completeness which no writer either medical or musical has before attempted.

After these explanations, the action of the parts in the medium register is more easily elucidated. In passing up the scale, the singer arrives at a point where the chest register becomes painfully laborious, he then changes to the medium register : immediately he is conscious of a diminution of pressure in his lungs, of some movement in his larynx, of a sudden sense of relief in the laryngeal muscles and of a greater smoothness in the quality of the tone. *The medium register is produced by partly relaxing the thyro-arytenoid muscles, so that the vocal chords shall not quite touch at their edges, but leave a narrow space between them.* In this state, it is obvious that they offer a less obstacle to passage of the air than when they are pressed together for the chest register; hence the diminished pressure in the lungs, of which the singer is conscious. The production of the voice does not necessarily require contact of the chords. In the artificial larynx which I have constructed, the best tones are produced when the ligaments are slightly separated. Draper, in his physiology, also states that contact of the chords is not requisite, although he does not recognize the bearing of the fact on the registers. In like manner, as I proved that in the chest register the chords are pressed together, I will now show that in the medium register they are separated. If the experimenter will deliver a tone on the medium register and gradually diminish the pressure of the lungs until the sound stops, being careful to maintain the laryngeal organs in the same position, he will find that though the breath is too feeble any longer to produce a sound, it will still escape with a faint hiss from the larynx, demonstrating beyond the possibility of error that the vocal chords are not in contact. The theory accounts beautifully for the sense of relief in the laryngeal muscles. When the thyro-arytenoids relax to allow the chords to fall apart, their relaxation diminishes at once the antagonism which they exerted against the crico-thyroids, and the latter having less of their force neutralized, are able with less effort to give the requisite tension to the vocal ligaments. This also is a point which, so far as I know, no writer has before explained. I suppose that the smoothness of the tone results from the fact that the separated chords no longer strike each other in their vibrations, and the smoothness of the sensation in the throat is for the same cause.

The head register of the female which is the falsetto of the male, is, as I said before, that flute like tone used by females on the highest notes, but which in male singers is condemned by teachers of music, because in them it is not of sufficient flexibility to repay cultivation. The proof that the falsetto of the male is a true head register is this: First, the quality of the tone is identical; secondly, boys before the age of puberty have a perfect head register like females. As the voice changes at puberty, the chest register is gradually extended downwards, while the head register is contracted in compass but not wholly lost, that which remains of it after the change of the voice being called falsetto.

The method in which the head tone is produced has never been explained. Draper says it is considered to be probably due to harmonic subdivisions of the column of air in the trachea, or to vibrations of the inner edge of the vocal chords. That this is not true, is evident from the fact that in wind instruments it is always the column of air *beyond* the point of sound, which regulates the pitch and not the column on the hither side of it. So of the vibration of the edges of the ligaments.—Their line of tension is parallel to the edge, therefore by the law of such organs, a vibration of the extreme edge alone would merely result in a feebler sound, but not in a higher pitch.

But if we examine more attentively the anatomy of the larynx, we find an arrangement exactly adapted to the production of the head register.

When a violinist wishes to obtain a high note from a string, he "stops" it against the finger board with his finger thereby shortening its vibratory part. Precisely analogous to this, there is an arrangement in the larynx for "stopping" the vocal chords so, as to render them virtually shorter. The anterior processes of the arytenoid cartilages are of considerable length, perhaps one fourth or one third of the entire anterior-posterior diameter of the larynx. Now if these cartilages are not firmly fixed, they will vibrate with the chords, and practically they are then a part of the chords, so that the vibratory organ includes not only the vocal tendon, but the processes of the arytenoids to which they are attached. This is the condition in the chest and medium registers, but in the head register I suppose that the arytenoids are placed in contact so firmly and so fixed by the muscles, that they no longer vibrate as a part of the cords, but by mutual pressure "stop" each other and become fixed points like pegs to which the tendons are fastened. The vibrations therefore are confined to the tendinous part of the chord, thus shortening them about one third, and rasing the pitch of the sound proportionally. Hence the reason why the highest notes can only be produced in the head register. With this shortening, the same amount of tension produces a much higher pitch than when the whole length of the organ vibrates according to well known acoustic laws, or conversely the same note can be given with much less muscular tension, hence the sense of relief which one feels in passing into this register.

So far as I know, no writer has ever before shown that the principle of "stopping" was made use of in the larynx. If so, I may claim to be the first one who has explained the physiology of the three registers of the human voice.—*Peninsular Journal.*

Chicago, Ill., Aug. 1, 1857.

E. Andrews.

